# Sensitive Immunochromatographic Assay Using Highly Luminescent Quantum Dot Nanobeads as Tracer for the Detection of Cyproheptadine Hydrochloride in Animal-Derived Food

**DOI:** 10.3389/fchem.2020.00575

**Published:** 2020-07-14

**Authors:** Pan Li, Cuifeng Yang, Beibei Liu, Qin Wu, Yulong Wang, Sa Dong, Hanxiaoya Zhang, Natalia Vasylieva, Bruce D. Hammock, Cunzheng Zhang

**Affiliations:** ^1^Key Laboratory of Food Quality and Safety of Jiangsu Province-State Key Laboratory Breeding Base, Key Laboratory of Control Technology and Standard for Agro-product Safety and Quality, Ministry of Agriculture, Institute of Food Safety and Nutrition, Jiangsu Academy of Agricultural Sciences, Nanjing, China; ^2^Tourism Department, Taiyuan University, Taiyuan, China; ^3^State Key Laboratory of Food Science and Technology, Nanchang University, Nanchang, China; ^4^College of Horticulture and Plant Protection, Yangzhou University, Yangzhou, China; ^5^Department of Entomology and Nematology and UCD Comprehensive Cancer Center, University of California, Davis, Davis, CA, United States

**Keywords:** immunochromatographic assay, quantum dot nanobeads, cyproheptadine hydrochloride, animal-derived foods, monoclonal antibody

## Abstract

Cyproheptadine hydrochloride (CYP), used as human and veterinary drug, has been used illegally as feed additive for food-producing animals, which could remain in food and jeopardize human health. There is a need for on-site detection of CYP residue in animal-derived food. In this study, a hapten was designed, and a specific monoclonal antibody (mAb) was developed to detect CYP with an IC_50_ of 1.38 ng/mL and negligible cross-reactivity (CR) for other analogs. Forthermore, a high sensitive immunochromatographic assay (QBs-ICA) was developed using quantum dot nanobeads as reporters. The assay showed the linear detection range (IC_20_-IC_80_) of 0.03–0.52 ng/mL, the limit of detection (LOD) and visual detection limit (VDL) reached to 0.01 and 0.625 ng/mL, respectively. Spiked recovery study in pig urine and pork confirmed that the QBs-ICA was applicable for on-site testing. This assay showed better sensitivity and speedy than the reported instrumental analysis and immunoassays.

## Introduction

Cyproheptadine hydrochloride (CYP), a derivative of cyproheptadine (CPH), is usually used as medicine to treat allergic disorders. Meanwhile, CYP has been prescribed as the drug for pet animals to stimulate appetite and induce weight gain, mostly for cats, dogs and horses (Gunja et al., 2004; Yamamoto et al., 2006; Rerksuppaphol and Rerksuppaphol, 2010). However, it has been found that CYP was illegally used as feed additive in food-producing animals to promote quick growth. After clenbuterol was banned for use in China, CYP and other derivatives of CPH have been frequently found in meat and dairy products, which may trigger allergic reactions or other sicknesses for the consumers (Yang et al., 2015; Kaur et al., 2018). Considering their risks for human health, CPH and CYP are also in the “List of Prohibited Drugs Used in Food-producing Animals” as stated in Directive 2001/82/EC (2009).

There is a need for the sensitive methods of the determination of CYP in animal food products. However, limited studies have been reported for on-site detection of CYP, regardless of the classic instrumental analysis or new immunoassays. Instrumental methods included molecularly imprinted solid-phase extraction coupled with high-performance liquid chromatography (HPLC) (Yang et al., 2015) and liquid chromatography-tandem mass spectrometry (LC-MS/MS) (Feás et al., 2009; Fente et al., 2009), which showed high accuracy and quantitative analysis properties. But there are inescapable realities that most instrumental methods are time-consuming and labor-intensive, which limit their use in on-site analysis or high-throughput screening for large numbers of samples. As complementary, immunoassays such as ELISAs are regarded as effective alternatives in terms of simplicity, speed and efficiency. However, they still require extended time for processing (Zhang et al., 2019; Zeng et al., 2020). Thereafter, immunochromatographic assay (ICA) was developed and used as a powerful on-site detection method. This assay is recognized for its outstanding speed, high specificity, sensitivity, low cost and user-friendliness. ICA is gaining increasing popularity as a diagnostic tool in food safety, animal health and the environment monitoring (Gong et al., 2017; Wang Z. et al., 2019; Lu et al., 2020). Colloidal gold (CG) nanoparticles as signal reporter are the most wildly used in ICA (CG-ICA), which provide obvious advantages in terms of detection cost, speed, long-term stability and with an easy distinct color readout (Li et al., 2020; Zeng et al., 2020). However, it does not provide advanced sensitivity comparisons with conventional ELISA. A alternative reporter, quantum dot nanobeads (QBs) could have been utilized to improve the detection sensitivity of ICA, because of their high quantum yield, broad UV excitation with narrow fluorescent emission spectra, large molar extinction coefficient, stronger fluorescence intensity and high photostability (Duan et al., 2015, 2019; Xiao et al., 2018; Chen et al., 2020). Wang et al. used QBs as luminescent amplification probes for ultrasensitive detection of tebuconazole in agricultural product and achieved a LOD of 0.02 ng/mL, which was more sensitive than other reports (Wang Y. et al., 2019). Duan et al. also developed QBs-ICA for quantitative detection of zearalenone in corns, the sensitivity was 5.6 times lower than the CG-ICA (Duan et al., 2015). Hu et al. established a QB-based sandwich ICA for the detection of C-reaction proteins, this assay was much more sensitive (257-fold) than CG-ICA (Hu et al., 2016).

In the current study, a hapten was designed and synthesized, which resulted in a high affinity antibody against CYP. Based on the resulting mAb, highly luminescent QBs were introduced in ICA as signal-amplification probes for CYP detection. This study provided a portable, user friendly, on-site detection approach which showed better sensitivity and speedy than the reported instrumental analysis and immunoassays (Fente et al., 2009; Yang et al., 2015; Guo et al., 2018).

## Materials and Methods

### Chemicals and Materials

CYP and loratadine, ketotifen, clenbuterol, clonidine and salbutamol (purity 99.0%) were obtained from Anpel Co., Ltd (Shanghai, China). Bovine serum albumin (BSA), dicyclohexylcarbodiimide (DCC), N,N-dimethylformamide (DMF), ovalbumin (OVA), trinbutylamine, isobutyl chlorocarbonate, N-hydroxysuccinimide (NHS), hypoxanthine-aminopterin-thymidine (HAT), complete and incomplete Freund's adjuvant, hypoxanthine-thymidine (HT) medium, peroxidase-labeled goat anti-mouse IgGs, 3,3′,5,5′-tetramethylbenzidine (TMB) and dimethyl sulfoxide (DMSO) were obtained from Sigma-Aldrich (St Louis, MO, USA). Nitrocellulose (NC) membranes, glass fiber sample pads and absorbent pad were purchased from Millipore Corporation (Bedford, MA, USA). Semi rigid polyvinyl chloride (PVC) sheets were obtained from Jiening Biotech (Shanghai, China). Quantum dots (QDs) were obtained in Prof. Yonghua Xiong's lab (Nanchang University, Nanchang, China). Dichloromethane (DCM), polyethylene glycol (PEG) 20000, poly (methyl methacrylate) (PMMA), poly (maleic anhydride-alt-1-octadecene) (PMAO), and other chemicals were obtained from Aladdin Chemistry Co., Ltd. (Shanghai, China).

### Equipment

ELISA absorbance readout was obtained by a multifunctional microplate reader from Thermo Fisher Scientific (Waltham, MA, USA). The XYZ3050 Dispensing Platform was purchased from BioDot (Irvine, CA, USA). The ZQ2000 Guillotine Cutter was purchased from Jinbiao Biotechnology (Shanghai, China). The fluorescence immunoassay analyzer was obtained from Helmen Co., Ltd. (Suzhou, China).

### Hapten and Antigen Synthesis and Characterization

The hapten was synthesized according to the synthetic route illustrated in Figure S1. Briefly, 5.8 g CYP and 2.73 g Et_3_N were dissolved in 100 mL DCM to obtain the crude product by drying, concentrating. Then the crude product and 11.4 g ClCO_2_Et were dissolved in 100 mL toluene to get a white solid by diluting and concentrating. 5.8 g white solid in EtOH was reacted with 13.7 g KOH in 22 mL water, and the mixture was extracted and washed to generate the white solid product. And then, 2.7 g product, 8.49 g methyl 2-bromoacetate and 1.4 g K_2_CO_3_ were dissolved in 27 mL DMF and stirred at 70°C for 16 h. After concentrating, 2.6 g product in 40 mL EtOH was reacted with 906 mg NaOH in 11 mL water at reflux for 1 h. After cooled, the solvent was removed, the mixture was adjusted pH to 2.0 with HCl. Finally, the mixture was purified, filtered to give hapten.

The immunogen (Hapten-BSA) was prepared as previously reported with some modifications (Feng et al., 2014). Briefly, 16.6 mg of hapten, 6.9 mg NHS and 12.36 mg DCC were dissolved in 1 mL DMF, and the mixture was stirred over night at room temperature (RT). Then, the precipitate was removed by centrifugation(12,000 g, 10 min), and 1 mL of the active ester was added dropwise into 5 mL of carbonate buffer (CBS, 100 mM, pH 9.6) containing 33 mg BSA, and the reaction mixture was stirred gently for 4 h. After dialyzed with phosphate buffer (PBS, 10 mM, pH 7.4), the immunogen was stored at −20°C.

Coating antigen (Hapten-OVA) was prepared using the mixed anhydride method (Liu et al., 2010). Briefly, 16.6 mg of hapten, 20 μL of trinbutylamine and 10 μL of isobutyl chlorocarbonate were mixed in 1 mL DMF with stirring for 3 h at 4°C. Then 1 mL mixture was added slowly into 5 mL of carbonate buffer containing 30 mg of OVA. After stirred, the product was dialyzed and stored at −20°C.

### Monoclonal Antibody Production and Characterization

Six–eight weeks old Balb/c female mice were immunized intraperitoneally with immunogen (Hapten-BSA). After the immunization, antisera were collected and were screened for anti-CYP activity by indirect competitive ELISA (ic-ELISA) (Cao et al., 2013). When the antisera showed the high anti-CYP activity, the last injection was performed intraperitoneally. Three days later, the cell fusion, and then subcloning procedures were carried out (Songa and Okonkwo, 2016). After several rounds of screening, a monoclonal cell strain with the good sensitivity for CYP recognition was selected and was used to prepare the ascites fluids. Then, the mAb was purified from ascites using a protein G affinity column. The ic-ELISA was used to characterize the mAb for CYP binding and cross-reactivity to analogs of CYP (Liang et al., 2013).

### Preparation of QBs

QBs were synthesized according to reported method (Duan et al., 2015). Briefly, 60 mg PMMA and 40 mg PMAO were dissolved with 1 mL of CHCl_3_. The 20 mg of CdSe/ZnS QDs and sodium dodecyl sulfonate aqueous solution (3 mg/mL; 5 mL) were added in mixture. Then, the mixture was emulsified using an ultrasonic homogenizer for 2 min. After the CHCl_3_ was evaporated from emulsion, the water-soluble QBs were purified by centrifugation (6,500 g, 10 min) and washed with pure water. The average size and morphology of QBs were characterized using a transmission electron microscope (TEM) with varying scales of magnifications.

### Preparation of the QBs-mAb

QBs-mAb was prepared according to reported method with slight modifications (Shao et al., 2018). Briefly, 5 μL of EDC (1 mg/mL) and 10 μL of QBs (12 mg/mL) were added to 2.7 mL of 0.01 M PBS (pH 6.0), then the mixture was magnetic stirred for 30 min at RT. And then the 100 μL mAb (1.2 mg/mL) was added, and the mixture was magnetic stirred for 30 min. Subsequently, the mixture was centrifuged at 12,000 g for 10 min, and the precipitates were resuspended with 400 μL of PBS containing 1% PEG 20000, 2% fructose, 5% sucrose, 1% BSA, and 0.4% Tween-20. And then the QBs-mAb probes were stored at 4°C. The QBs-mAb and free QBs probe were analysis with Dynamic light scattering (DLS).

### Fabrication of Immunochromatographic Test Strips

First off, the sample pads and absorbent pads were pretreated by soaking with 10 mM PBS (pH 7.4) containing 0.02% NaN_3_, 2% BSA, 2.5% sucrose and 0.25% Tween-20, and these pads were dried at 37°C overnight. Then, NC membrane was pasted onto a backing card. Goat anti-mouse antibody and Hapten-OVA were coated on NC membrane as control (C) lines and the test (T) lines, respectively. The distance between the C line and the T line is 7 mm. Subsequently, the dried sample pads and absorbent pads were pasted onto a backing card, and laminated with overlaps of 2–3 mm at bottom and top edge of NC membrane, respectively. Then, the plate was cut lengthwise into strips (3.5 × 60 mm) and stored in dry places (Liu et al., 2018).

### Assay Procedure

1 μL of QB-mAbs probe and 70 μL of standard solution (diluted with PBS containing 5% methanol) were premixed for 5 min and added into the sample pad. After 10 min, the fluorescence intensities of T lines (FI_T_) and C lines (FI_C_) were recorded with a fluorescence analyzer. The standard curve was constructed by plotting the B/B_0_ × 100% against the logarithm of the CYP concentration, where B represented FI_T_/FI_C_ with CYP standard solutions and B_0_ represented FI_T_/FI_C_ without the presence of CYP standard solutions (Shao et al., 2018; Wang Y. et al., 2019).

### Spiked Samples

The pig urine and pork samples were CYP free as determined by confirming with instrumental analysis. Test samples were spiked with known concentrations CYP.

For QBs-ICA analysis, 20 mL CYP spiked pig urine samples were centrifuged at 10,000 g for 15 min. Then the supernatant was diluted 5-fold with PBS containing 5% methanol. 5.0 g homogenized pork samples were added into 50 mL of acetonitrile containing 20% of ammonia. And then the mixture was stirred vigorously for 15 min, and further centrifuged at 10,000 g for 15 min. The 10 mL supernatant was added to 10 mL of n-hexane, shaken vigorously to let the phases separate, the lower acetonitrile phase was saved and evaporated to dryness. Finally, the dry residue was redissolved with 1 mL PBS containing 5% methanol, and further diluted 5-fold with PBS containing 5% methanol for QBs-ICA analysis (Liu et al., 2010).

For instrumental method analysis, the extracts of pig urine and pork samples were evaluated by liquid chromatography-tandem mass spectrometry (LC/MS-MS) according to the previous report with slight modification (Fente et al., 2009; Sun et al., 2017). In the liquid chromatography (LC) system, C18 column (2.1 × 150 mm, 2.7 μm) was employed for separation, using acetonitrile and 10% formic acid (80%: 20%, v/v) as carrying mobile phase. The mobile phase was running at 0.35 mL/min and maintaining the column temperature at 30°C, and the injected volume of extracts in the column was 5 μL. For the mass spectrometric analysis, the electrospray ionization source was operated at 150°C in the positive ion mode. Multiple reaction monitoring (MRM) mode was used, and the optimized parameters for mass detection are referred to Fente's report (Fente et al., 2009).

## Results and Discussion

### Characterization of Hapten and Antigen

The molecular structure of the hapten was determined by LC/MS (Figure S2) and ^1^HNMR (Figure S3). LC/MS (ESI) calcd for C_22_H_21_NO_2_: 331.16. Found: *m/z* 332.1 [M+H]^+.^. The results of ^1^HNMR confirmed the hapten has successfully attached to a carboxyl group: δ: 7.40–7.36 (m, 4H), 7.30–7.26 (m, 2H), 7.21–7.19 (m, 2H), 6.97 (s, 2H), 3.12 (s, 2H), 2.78–2.76 (m, 2H), 2.54–2.43 (m, 2H), 2.35–2.30 (m, 2H), 2.06–2.03 (m, 2H).

As a small molecule, CYP cannot trigger immune response. Therefore, CYP was conjugated to a carrier protein of BSA or OVA to be used as immunogen and coating antigen, respectively. As illustrated in the UV-Vis spectra (Figure S4), the absorption of immunogen (Hapten-BSA) and coating antigen (Hapten-OVA) shifted in comparison with the hapten and the carrier protein, that supported the successful conjugation of the CYP and carrier proteins.

### Characterization of mAb

The mAb were purified from ascites using a protein G affinity column. Thereafter, ic-ELISA method was conducted for characterization. The working concentration of the coating antigen and mAb were 0.375 μg/mL and 0.15 μg/mL, respectively. In the linear range (IC_20_-IC_80_) of 0.31–6.03 ng/mL, the half-maximal inhibitory concentration (IC_50_) was 1.38 ng/mL, the limit of detection (LOD) was 0.13 ng/mL. Loratadine, ketotifen, clenbuterol, clonidine and salbutamol as the analogs of CYP were used in specificity test, the cross-reactivity (CR) values were <0.01% (Table 1) of the CYP response.

**Table 1:** Cross-reactivity against CYP and other analog compounds by ELISA.

**Chemicals**	**Structure**	**IC_**50**_ (ng/mL)**	**CR (%)**
CYP	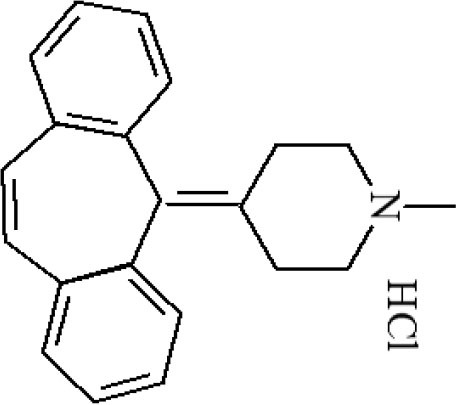	1.38	100
Loratadine	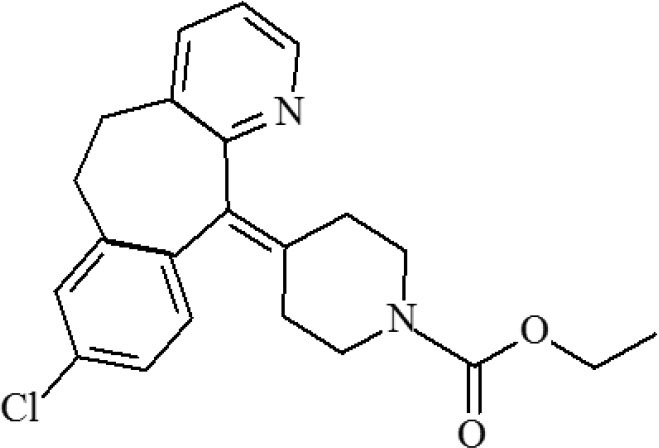	>100	<0.01
Ketotifen	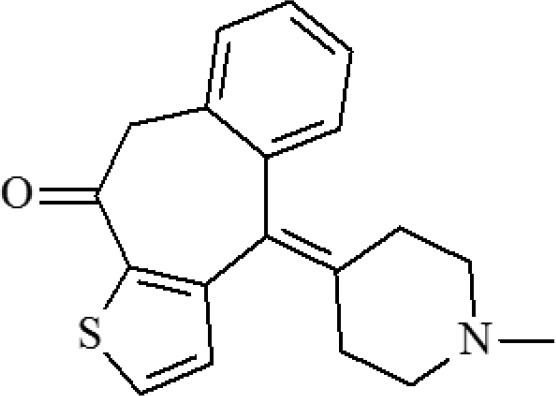	>100	<0.01
Clenbuterol	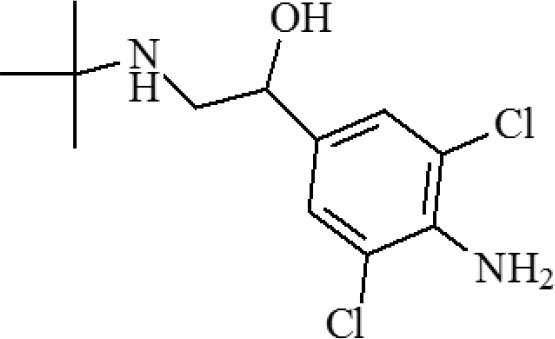	>100	<0.01
Clonidine	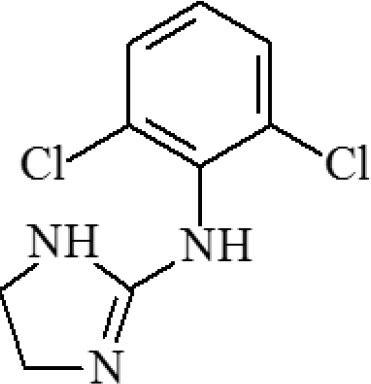	>100	<0.01
Salbutamol	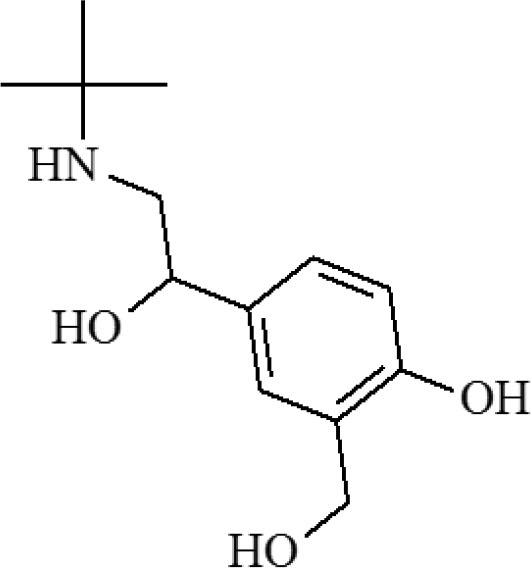	>100	<0.01

### Characterization of QBs and QBs-mAb Conjugates

**Figure 1:**
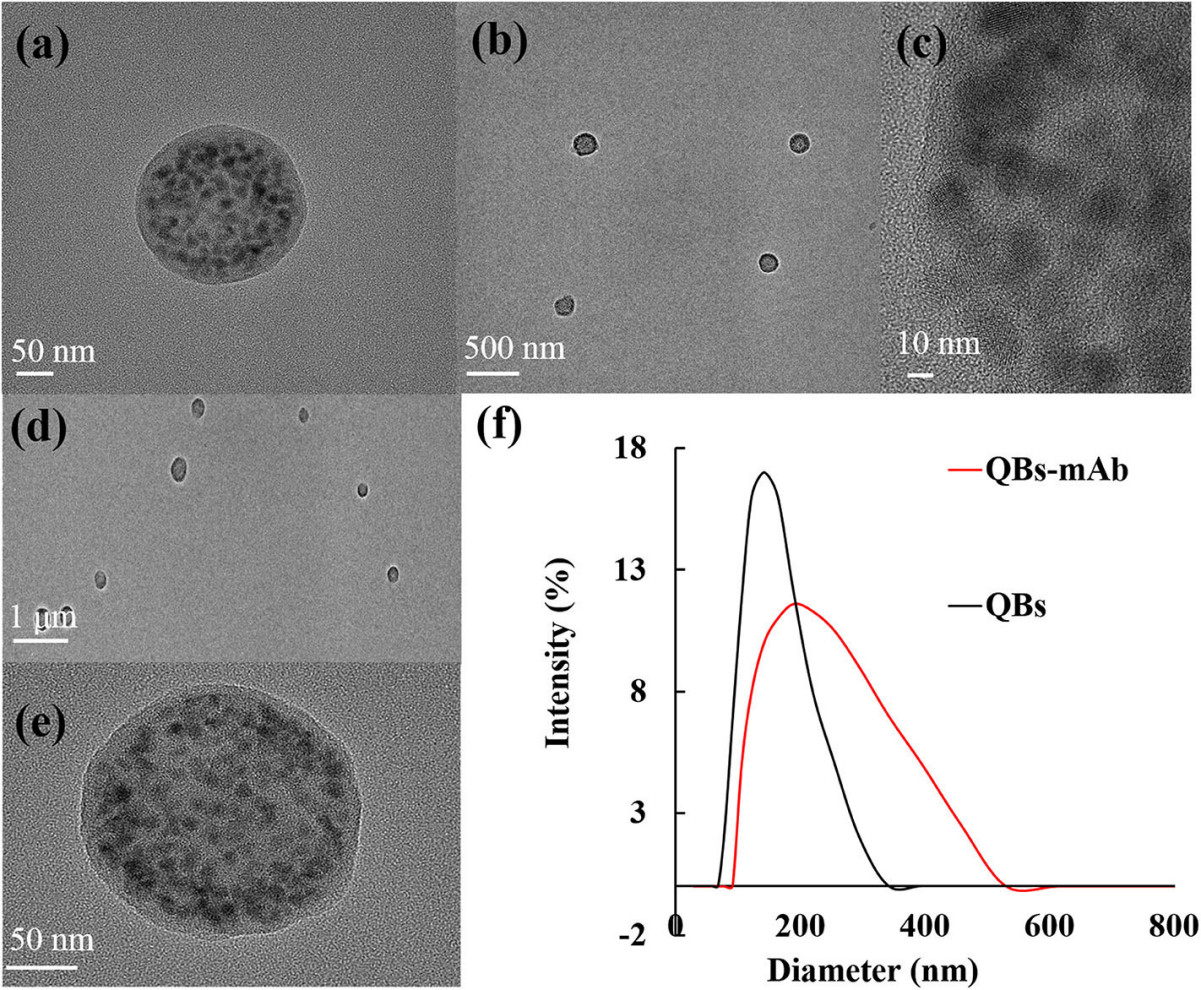
Characterization of QBs and QBs-mAb. **(a–c)**: TEM image of the QBs at different magnification; **(d–e)** TEM image of the QBs-mAb at different magnification **(f)**: Hydrodynamic diameter of QBs and QBs-mAb.

The QBs and QBs-mAb were monitored using a TEM with varying scales of magnifications. As Figure 1a shows that the numerous dark QDs are tightly encapsulated in the polymer matrix. Figure 1b shows that the QBs have a relatively uniform size distribution. Figure 1c also supports a macroscopic metal lattice structure of CdSe/ZnS QDs. The QBs-mAb probes were characterized with TEM and DLS. Figures 1d,e show the morphology of probes are similar as the QBs's. Figure 1f shows the hydrodynamic diameters of free QBs is 142 nm, and after the anti-CYP mAbs were conjugated on the surface of the QBs, the hydrodynamic diameters increased to 190 nm. The results support that the anti-CYP mAbs were successfully coupled on the surface of the QBs.

### ICA Testing

**Figure 2:**
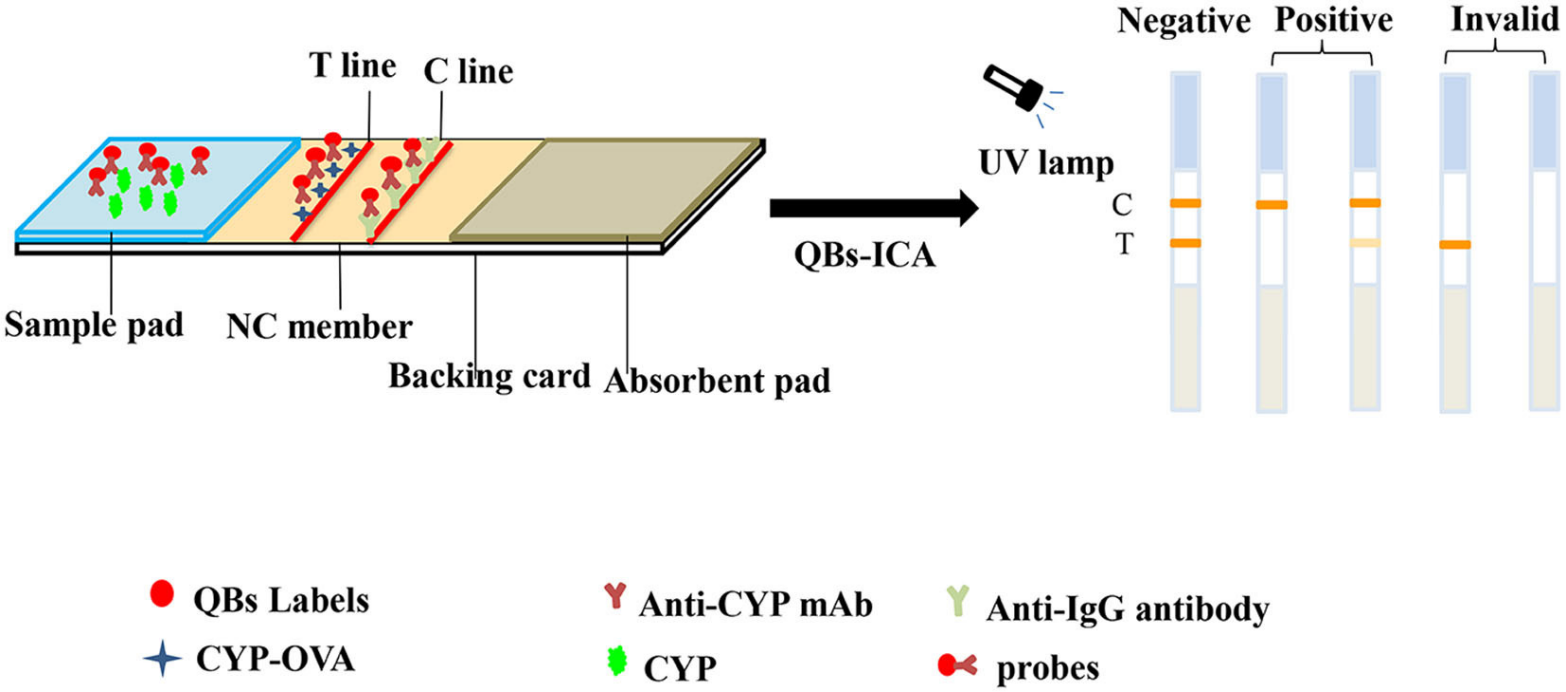
Schematic diagram of QBs-ICA testing.

**Figure 3:**
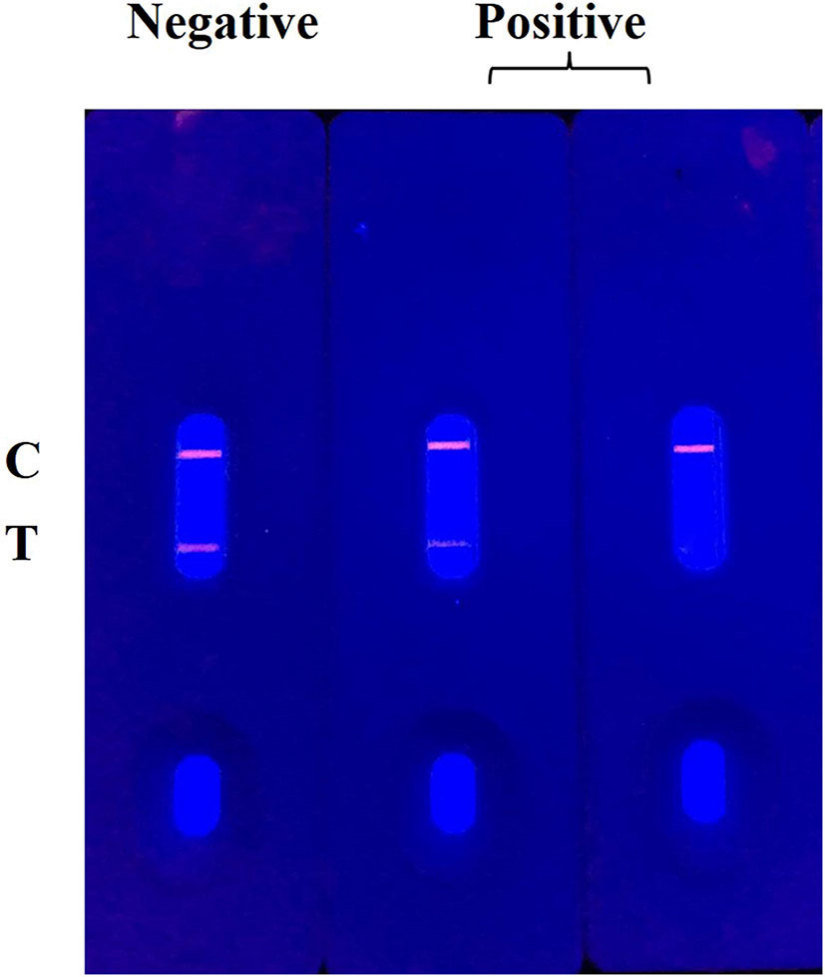
The result of QBs-ICA testing.

The principle and result of the QBs-ICA strip testing are illustrated in Figures 2, 3. In absence of CYP, the labeled mAbs were captured by the immobilized hapten conjugates on the T line, and the excess labeled mAb migrated further to interact with goat anti-mouse IgG on the C line. For negative samples, the fluorescence on the T line and C line was similar showing a negative result. In presence of CYP, the labeled mAbs combined with the CYP and migrated over the membrane without (or in less extend) interaction on the T lines. The FI_T_ is lower than the negative control or even invisible, this indicates a positive result. So, the FI_T_ was reversely correlated with the concentration of analytes (Foubert et al., 2017). The C line showed always be visible as an ICA quality indicator, otherwise the ICA should be considered in error. In addition, for on-site detection using QBs-ICA, a portable UV-based analyzer or UV lamp is needed.

### Optimization of QBs-ICA

To achieve the best performance and sensitivity of the immunoassay, the appropriate content of mAb, QBs-mAb probe and Hapten-OVA employed were crucial. Content of mAb labeling on the surface of the QBs were estimated by using a series of mAb (2–14 μL) to conjugate 1 μL of QBs, and measured by the change of fluorescence intensity. The QB-mAbs conjugates were assembled and tested on the strips, the FI_T_ were recorded by an analyzer. Figure S5A demonstrates that FI_T_ increased drastically along with the continuous addition of mAbs before reaching a plateau at 10 μL. These data suggested that 10 μL of mAbs (1.2 mg/mL) per μL of QBs (12 mg/mL) was considered as the saturated labeling concentration.

For the best sensitivity and appropriate FI signal, the contents of QBs-mAb probe upon Hapten-OVA were optimized with a “checkerboard titration-like” tactic (Guo et al., 2019). The inhibition rate (IR) of CYP at 0.3 ng mL^−1^ are obtained by the formula: *IR* = (1−*B*/*B*_0_) × 100%, where B represented FI_T_/FI_C_ with CYP presence and B_0_ represented FI_T_/FI_C_ with the absence of CYP. As shown in Table S1, the highest IR was 61%, while 1.0 mg/mL of the Hapten-OVA were immobilized on the test line, and the QBs-mAb probe was 1 μL, respectively.

In addition, for the stability and sensitivity optimization of QBs-ICA, an immunological dynamic analysis was conducted to optimize the interpretation time and pH value (Huang et al., 2013). The kinetic curves were established by recording FI_T_, FI_C_, every 1 min for 30 min to calculate FI_T_/FI_C_ ratio. As shown in Figure S5B, FI_T_, FI_C_ continue to increase from 1 to 30 min, but the FI_T_/FI_C_ ratio tended to stabilize after 10 min. That means at least a 10 min reaction time was necessary for good QBs-ICA performance.

The effect of pH values on sensitivity of QBs-ICA was evaluated by adjusting the pH values of the sample solutions to 5.0, 5.5, 6.0, 6.5, 7.0. This allowed exploration of pH on FI_T_/FI_C_ and IR for CYP determination at the concentration of 0.3 ng/mL. The results as shown in Figure S5C, the FI_T_/FI_C_ of negative sample had no significant decrease from pH 5.0 to 6.0, and the maximum IR of 69% emerged in pH 6.0. When pH values ranged from 6.0 to 7.0, the FI_T_/FI_C_ of negative sample and IR decreased sharply. Thereby, the optimal pH condition was chosen as 6.0 for QBs-ICA performance in this case.

The methanol would also effects the sensitivity of QBs-ICA, but a certain amount of methanol is required to assist the dissolution of the CYP. In this study, it was found that the methanol content less to 5% with negligible effect.

### Sensitivity of the QBs-ICA

**Figure 4:**
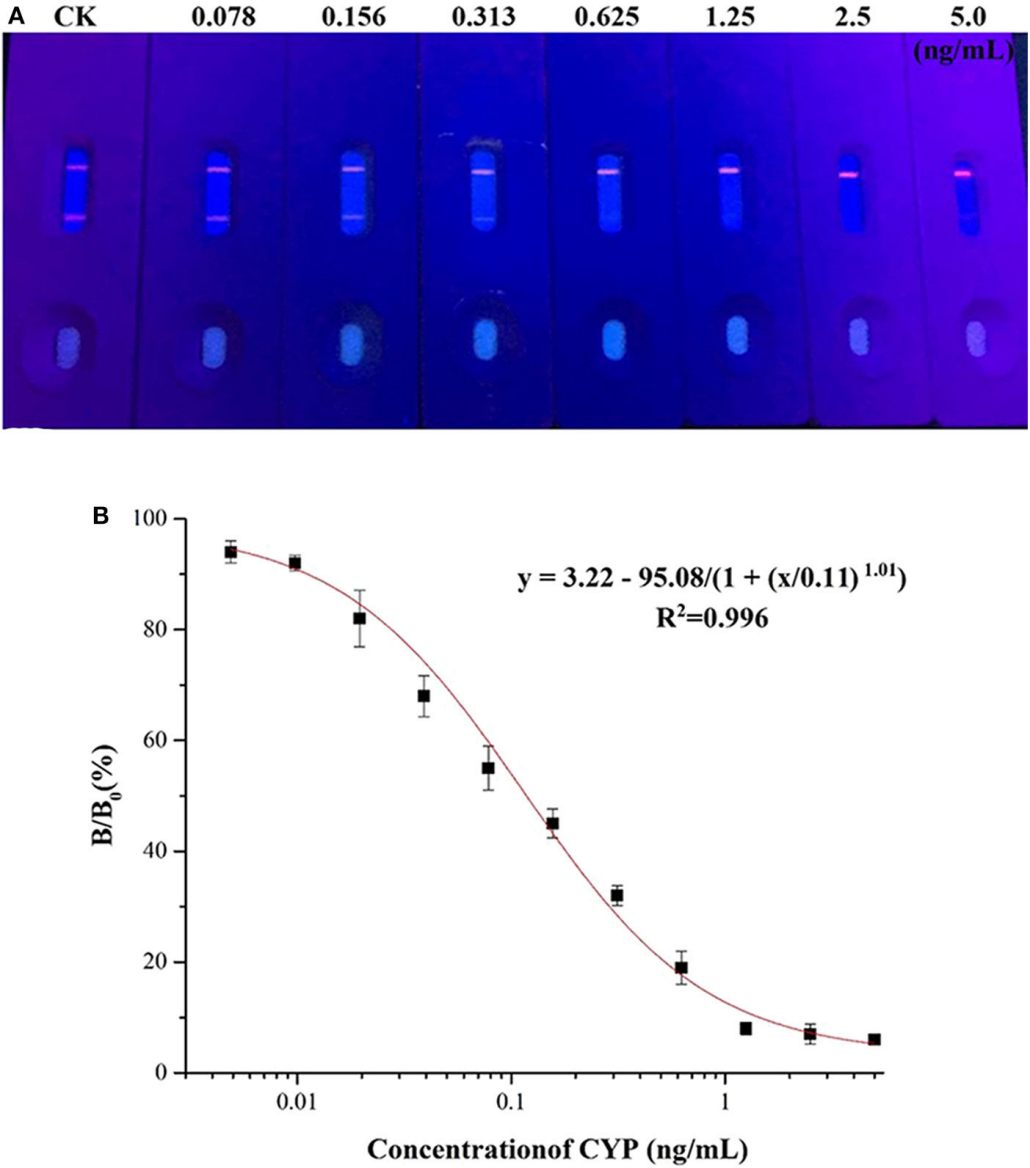
Sensitivity of the QBs-ICA. **(A)** Photo of the test strips with different CYP concentrations (0.078–5.0 ng/mL) under 365 nm UV excitation. **(B)** Standard competitive inhibition curve for CYP under the optimized conditions.

A series of CYP solutions were prepared with working buffer (10 mM PBS with 5% methanol, pH 6.0) to investigate the sensitivity of the QBs-ICA. As Figure 4A illustrates the fluorescence on the T line gradually decreased as the concentration of CYP increased, and finally it disappeared at 0.625 ng/mL. This suggested that the visual detection limit (VDL) is about 0.625 ng/mL for visual discrimination and semi-quantitative detection. Furthermore, the quantitative analysis of QBs-ICA can be calculated by plotting the B/B_0_ × 100% against the logarithm of the CYP concentration, where B represented FI_T_/FI_C_ in the presence of CYP, and B_0_ represented FI_T_/FI_C_ in the absence of CYP. Figure 4B illustrates the standard curve of QBs-ICA for CYP testing, the IC_50_ and LOD (IC_10_) values were 0.12 and 0.01 ng/mL, respectively. The linear range (IC_20_-IC_80_) was 0.03–0.52 ng/mL. Compared to previously reported, the QBs-ICA shows superior performance in terms of both sensitivity and speed of analysis than the only reported immunoassays (about 10-fold improvement) (Guo et al., 2018) and the instrumental analysis (about 2–3 orders of magnitude improvement) (Fente et al., 2009; Yang et al., 2015), the comparation was shown in Table 2.

**Table 2:** Comparison of different methods for the detection of CYP.

**Analysis method**	**Assay sample**	**LOD (ng/mL)**	**Time** **(min)**	**VDL^b^** **(ng/mL)**	**References**
HPLC	feed	40	>60	ND^a^	(Yang et al., 2015)
LC-MC/MC	pig urine	0.48	>60	ND^a^	(Fente et al., 2009)
GC-ICA	pig urine	ND^a^	15	5	(Guo et al., 2018)
QBs-ICA	pig urine, pork	0.01	10	0.625	This work

a *Not detected*.

b *Visual detection limit*.

### Specificity of the QBs-ICA

**Figure 5:**
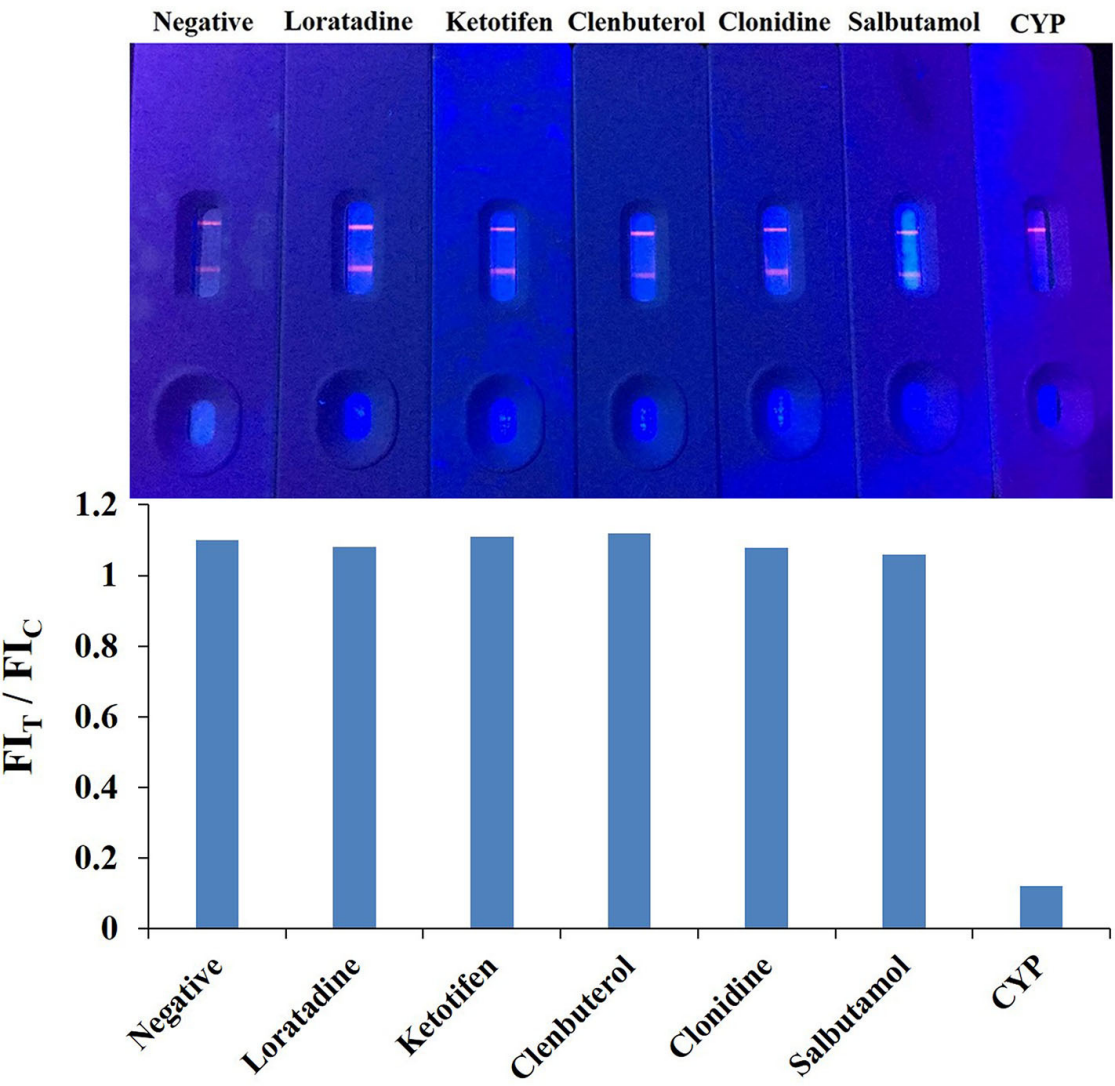
Cross-reactivity study of QBs-ICA against CYP (1 ng/mL) and other analogs (1 μg/mL).

The specificity of the QBs-ICA was tested with CYP and five other analogs. As Figure 5 shows, compared with the negative control, the fluorescence on the T line for CYP (1 ng/mL) had completely disappeared, while the fluorescence of T lines of five analogs (1 μg/mL) barely dropped of, and the values of FI_T_/FI_C_ for five analogs almost as the same as negative control. This means that the antibody does not recognize these analogs, and the CRs were negligible. The results indicated that the high specificity of QBs-ICA for CYP detection, which also consistent with the results of cross-reactivity using ic-ELISA (Li et al., 2019).

### Method Validation by Spiked Samples

For LC/MS-MS, chromatographic separation was achieved (Figure S6), in that, the good efficiency and peak shape were obtained in a 1.4 min analysis time.

Matrix interferences is a common challenge for immunoassay, and it may lead to false positive or negative results and reduce the sensitivity and reliability of the methods (Hua et al., 2012). In this study, spiked pig urine, pork samples were used to evaluate the feasibility of the developed QBs-ICA method. It showed that the matrix interferences were reduced by a 5-fold dilution. As summarized in Table 3, the recoveries ranged from 85 to 105% and the coefficient of variation (CV) were <11%. The results of QBs-ICA were consistent with LC/MS-MS data, suggesting that the developed QBs-ICA was accurate and practicable for CYP detection in animal food. In conclusion, the developed QBs-ICA are reliable and sensitive to be further used for CYP analysis.

**Table 3:** Results of QBs-ICA and LC-MS/MS for spiked samples (pig urine and pork).

		**QBs-ICA**			**LC-MS/MS**		
**Sample**	**Fortified (ng/mL) or (ng/g)**	**Mean (ng/mL) or (ng/g)**	**Recovery (%)**	**CV** **(%)**	**Mean (ng/mL) or (ng/g)**	**Recovery (%)**	**CV** **(%)**
Pig urine	0.5	0.425	85	3.1	0.45	90	1.2
	2	2.1	105	11	1.96	98	7.9
	5	4.85	97	2.5	4.75	95	3.6
Pork	0.5	0.445	89	1.2	0.47	94	4.4
	2	1.96	98	5.0	2.1	105	7.4
	5	4.75	95	1.0	4.9	98	4.7

## Conclusions

Very few studies have been reported for the on-site detection of CYP residues in animal products. In this paper, we developed a QBs-ICA for the rapid and on-site detection of CYP in pig urine and pork samples. A specific monoclonal antibody (mAb) against cyproheptadine hydrochloride (CYP) was induced using designed hapten. Thereafter, QBs-ICA was developed using quantum dot nanobeads as indicators for visual and quantitative detection of CYP. Spiked sample study confirmed that the QBs-ICA was capable of being used as portable and rapid tools for on-site testing. Under optimal conditions, the developed QBs-ICA had visual detection limit of 0.625 ng/mL, the sensitivity and speedy were much improved compared with the reported instrumental analysis and immunoassays (Fente et al., 2009; Yang et al., 2015; Guo et al., 2018).

## Data Availability Statement

All datasets presented in this study are included in the article/Supplementary Material.

## Ethics Statement

The animal study was reviewed and approved by Jiangsu Academy of Agricultural Sciences.

## Author Contributions

PL: carried out experiments, analyzed data, and wrote the manuscript draft. CY: carried out experiments and analyzed data. BL and QW: assisted with experiments. YW and SD: gave experimental guidance. CZ: designed the experiments and analyzed data. BH, HZ, and NV: revised the manuscript. All authors contributed to the article and approved the submitted version.

## Conflict of Interest

The authors declare that the research was conducted in the absence of any commercial or financial relationships that could be construed as a potential conflict of interest.
