# Supplementary material for: Sensitive Immunochromatographic Assay Using Highly Luminescent Quantum Dot Nanobeads as Tracer for the Detection of Cyproheptadine Hydrochloride in Animal-Derived Food
Source: Front Chem. 2020 Jul 14;8:575. doi: 10.3389/fchem.2020.00575 (PMC7372008; doi:10.3389/fchem.2020.00575)
Supplement: Supplementary file 1 [file Table_1.doc]

***Supporting information***

**Sensitive immunochromatographic assay using highly luminescent quantum dot nanobeads as tracer for the detection of cyproheptadine hydrochloride in animal-derived food**

Pan Li^a,1^, Cuifeng Yang^b,1^, Beibei Liu^a^, Qin Wu^a,c^, Yulong Wang^a^, Sa Dong^a,d^, Hanxiaoya Zhang^a^, Natalia Vasylieva^e^, Bruce D. Hammock^e^, Cunzheng Zhang^a,c^*

*^a^ Key Lab of Food Quality and Safety of Jiangsu Province-State Key Laboratory Breeding Base, Key Laboratory of Control Technology and Standard for Agro-product Safety and Quality, Ministry of Agriculture, Institute of Food Safety and Nutrition, Jiangsu Academy of Agricultural Sciences, Nanjing 210000, China*

*^b^ Tourism department, Taiyuan University, 030032 Taiyuan, Shanxi Province, PR China.*

*^c^ State Key Laboratory of Food Science and Technology, Nanchang University, Nanchang 330000, China*

*^d^ College of Horticulture and Plant Protection, Yangzhou University, Yangzhou 225000, China*

*^e^ Department of Entomology and Nematology and UCD Comprehensive Cancer Center, University of California, Davis, California 95616, United States*

** Corresponding authors E-mail addresses: zhcz2003@hotmail.com*

*^1^ Pan Li and Cuifeng Yang have equal contribution to the paper*

**Fig. S1** Synthesis schematic of hapten.

**Fig. S2** Positive ion electrospray mass spectra of hapten.

**Fig. S3** ^1^H-NMR spectra of hapten measured in DMSO at 400 MHz.

**Fig. S4** UV-Vis spectra of hapten, carrier protein and conjugate.

**Fig. S5** Optimization of the QBs-ICA. (a) Fluorescence varies in the changing concentrations of anti-CYP mAb (1.2 mg/mL) on the surface of QBs. (b) Immunoreaction dynamics of FI_T_, FI_C_, and FI_T_/FI_C_ ratio upon time. (c) The impacts of pH on the FI_T_/FI_C_ and inhibition rate (IR) for CYP.

**Fig. S6** LC-MS/MS chromatograms of CYP (spiked 100 ng/mL).

**Table S1** Optimization of the concentrations of Hapten-OVA and QBs-mAb probe using a checkerboard titration.

**
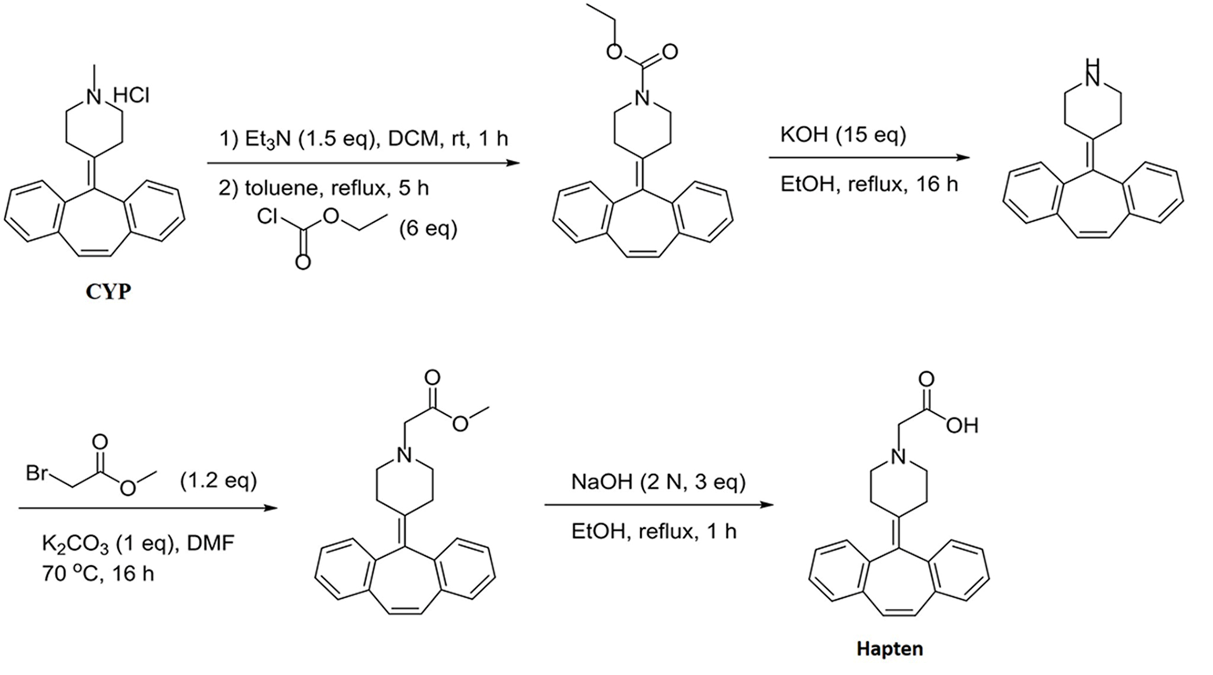
**

**Fig. S1** Synthesis schematic of hapten.


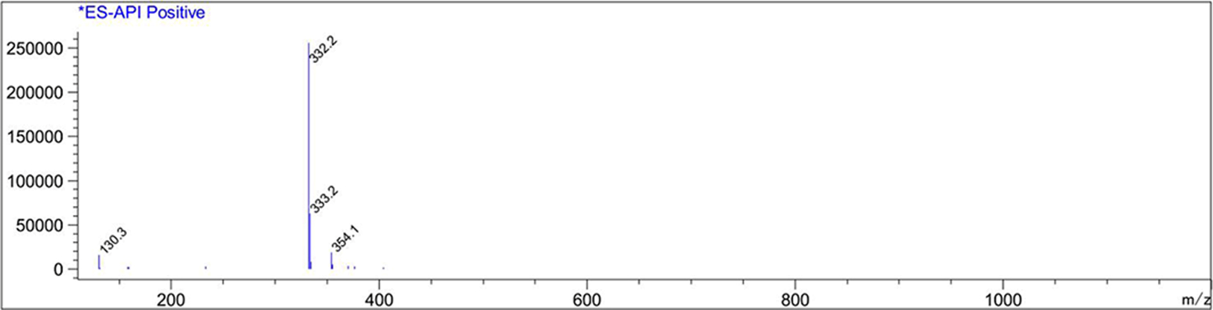


**Fig. S2** Positive ion electrospray mass spectra of hapten.


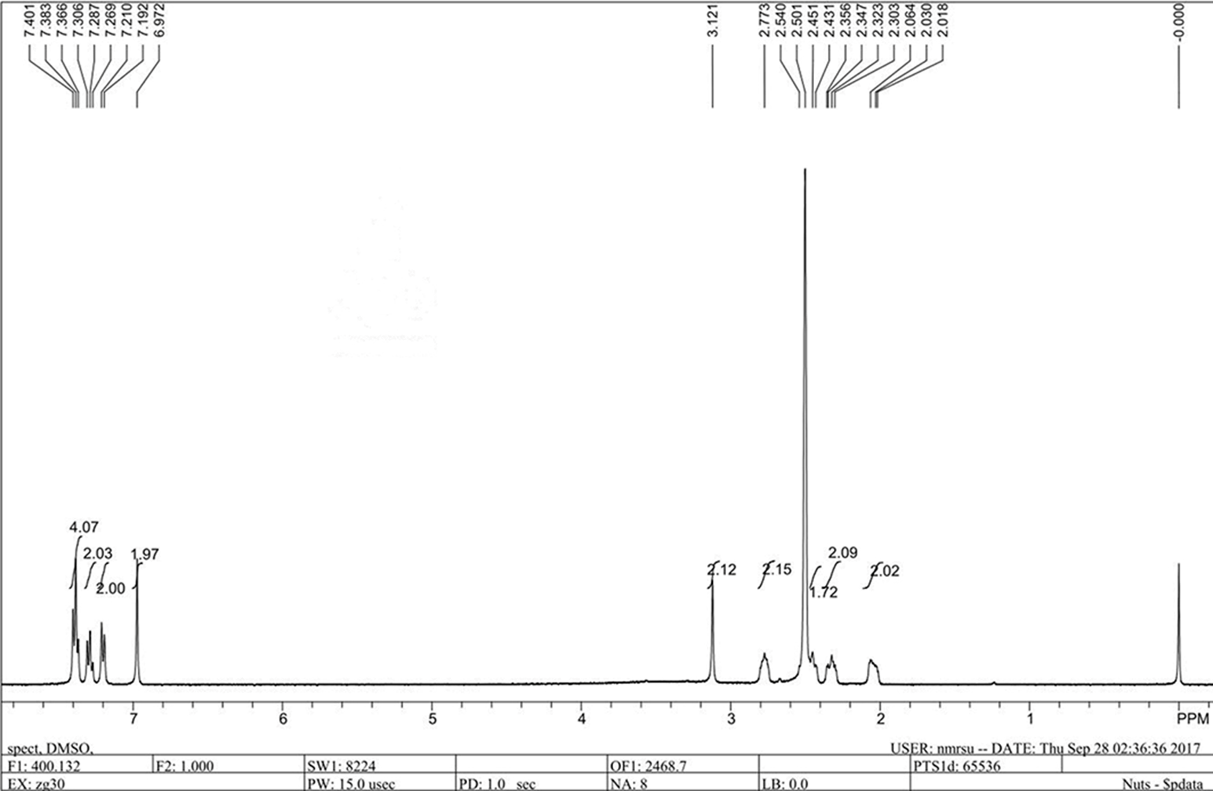


**Fig. S3** ^1^H-NMR spectra of hapten measured in DMSO at 400 MHz.


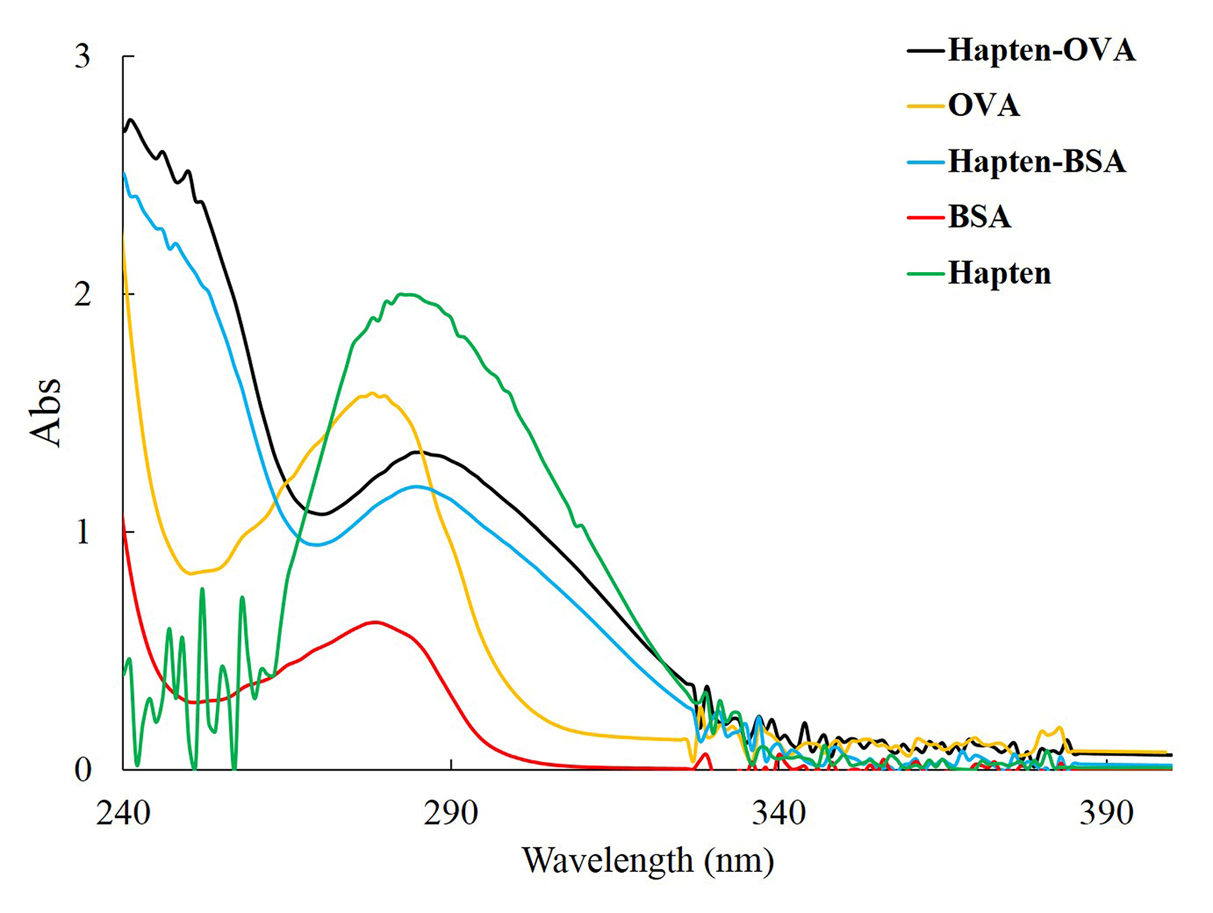


**Fig. S4** UV-Vis spectra of hapten, carrier protein and conjugate.


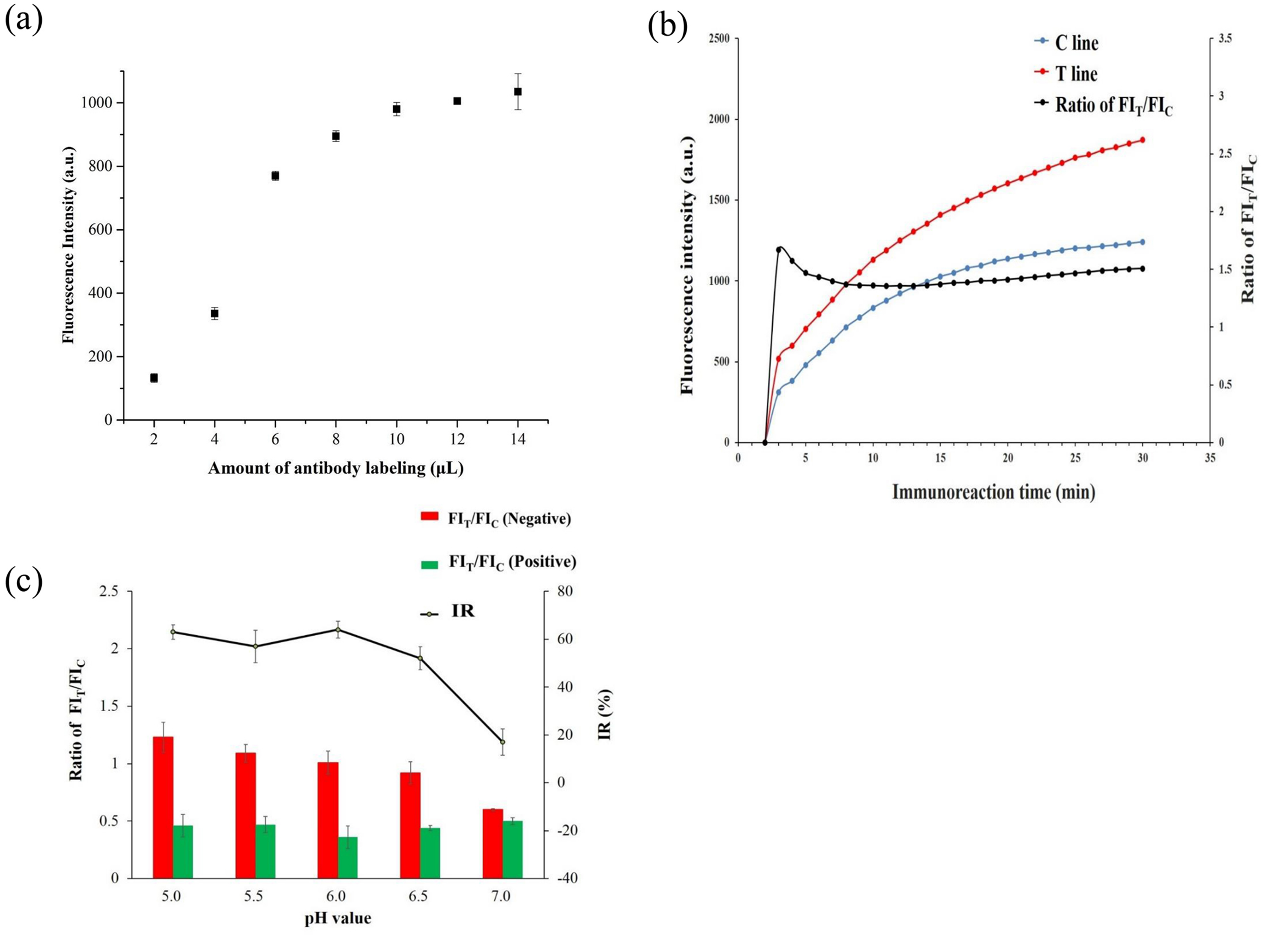


**Fig. S5** Optimization of the QBs-ICA. (a) Fluorescence varies in the changing concentrations of anti-CYP mAb (1.2 mg/mL) on the surface of QBs. (b) Immunoreaction dynamics of FI_T_, FI_C_, and FI_T_/FI_C_ ratio upon time. (c) The impacts of pH on the FI_T_/FI_C_ and inhibition rate (IR) for CYP.


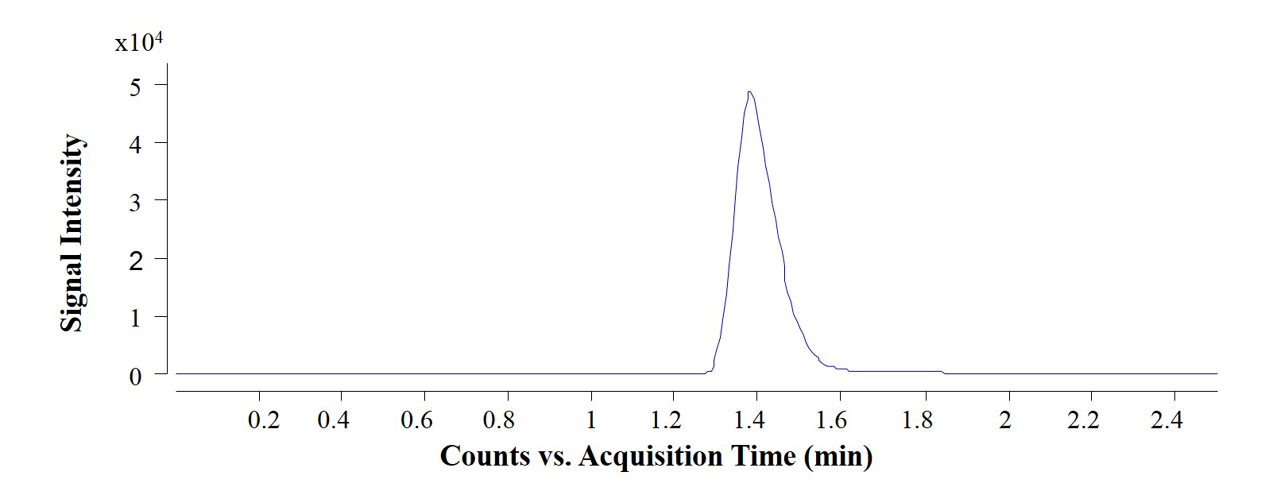


**Fig. S6** LC-MS/MS chromatograms of CYP (spiked 100 ng/mL).

**Table S1** Optimization of the concentrations of Hapten-OVA and QBs-mAb probe

using a checkerboard titration.

| No. | Hapten-OVA  (mg/mL) | QBs mAb  (µL) | FI_T_^a^ | FI_C_^a^ | IR (%)^b^ |
| --- | --- | --- | --- | --- | --- |
| Group 1  Group 2  Group 3 | 0.8  0.8  0.8  1.0  1.0  1.0  1.2  1.2  1.2 | 1  2  3  1  2  3  1  2  3 | 550  712  887  798  900  1180  550  712  887 | 523  689  812  768  891  1123  917  1011  1213 | 31  40  45  61  51  41  41  45  37 |
